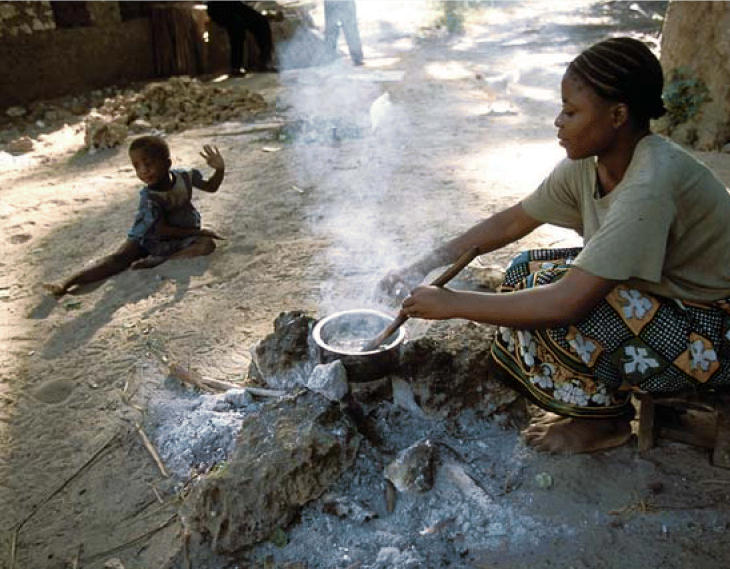# Building Research Capacity in Developing Nations

**Published:** 2006-10

The NIEHS Strategic Plan for 2006–2011, *New Frontiers in Environmental Sciences and Human Health,* lays out a vision for establishing a global effort in the environmental health sciences. A central component of that vision is the establishment of enhanced research capacity in developing nations and cooperation between the research communities here in the United States and abroad. To that end, we have announced a new program to bring junior-level researchers from developing nations for short-term research experiences in NIEHS-funded labs, and we have expanded our existing programs in cooperation with the Fogarty International Center.

## Supplements to Support Research Capacity in Developing Nations

The highlight of this effort is a new administrative supplement program intended to provide an opportunity for junior faculty–level scientists from developing nations to conduct research in NIEHS-funded laboratories. This opportunity is available to principal investigators with NIEHS-funded R01, R37, or P01 awards and provides salary and travel support for the foreign investigator. Applications are considered on a rolling basis and must include a description of the research project to be conducted by the foreign investigator, a mentoring plan, and a career goals statement. Additional detailed information is available on the web at **http://www.niehs.nih.gov/dert/programs/capacity.htm**.

## Fogarty International Research Collaboration Award (FIRCA)

An additional effort is being made to build research capacity in these developing nations through the NIEHS’s continued participation in the FIRCA basic biomedical research program (**http://www.fic.nih.gov/programs/research_grants/firca/index.htm**). The FIRCA grant mechanism allows the development of collaborative programs between established environmental health researchers and investigators who remain in their developing countries. This opportunity includes both laboratory- and population-based studies on the biological response to environmental exposures to pollutants as well as studies of susceptibility factors due to genetics, age, nutritional status, or co-morbid conditions. The NIEHS is currently co-funding 17 grants with the Fogarty International Center under the FIRCA program.

## Contacts

**Dr. William Suk, Ph.D.** | suk@niehs.nih.gov

**Dr. David Balshaw, Ph.D.** | balshaw@niehs.nih.gov

## Figures and Tables

**Figure:**